# Was the Military Operation “Protective Edge” a Risk Factor for Pregnancy Complications?

**DOI:** 10.5041/RMMJ.10304

**Published:** 2017-04-28

**Authors:** Ohad Gluck, Yossi Mizrachi, Sophia Leytes, Jacob Bar, Michal Kovo

**Affiliations:** Department of Obstetrics and Gynecology, E. Wolfson Medical Center, Holon, Israel, and Sackler School of Medicine, Tel Aviv University, Tel Aviv, Israel

**Keywords:** Mental stress, pregnancy complications, preterm labor

## Abstract

**Objective:**

During July–August 2014, the military operation “Protective Edge” presented Israel with a threat of missile attacks. We aimed to investigate the influence of the “Protective Edge” operation on the rate of pregnancy complications among the population exposed to missile attacks, compared to the population not exposed.

**Study Design:**

This was a retrospective study. Pregnancy outcomes were compared between women who during pregnancy were exposed to the stress of the military operation (exposed group, *n*=4,673) and gave birth at the Wolfson Medical Center, and women who gave birth in the previous year (unexposed group, *n*=4,735).

**Results:**

Rates of pregnancy complications did not differ between the groups.

**Conclusion:**

Exposure to environmental stress during pregnancy, for a period of almost two months, was not found to be associated with increased risk for pregnancy complications.

## INTRODUCTION

Maternal stress during pregnancy has been associated with adverse birth and pregnancy outcomes. Studies have demonstrated an association between maternal mental stress during pregnancy and pregnancy complications, including low birth-weight, preterm delivery, and preeclampsia, especially among highly exposed women.^1^–^5^

Several mechanisms have been proposed for explaining this association, and the most widely accepted theory is based on the hypothalamic–pituitary–adrenal (HPA) axis response to stress. The activation of the HPA axis, as part of the stress mechanism, leads to an increased secretion of cortisol and catecholamines, which results in blood pressure elevation, blood glucose levels rising, and continuous oxidative stress.^3^

Furthermore, during pregnancy there is a gradual increase in cortisol level, and it peaks before delivery. Chronic stress during pregnancy may elevate blood cortisol level prematurely, and by that lead to preterm delivery. In addition, catecholamine secretion, as a response to stress, may induce preterm contractions, which could lead to preterm delivery.^6^

The influence of military conflicts on pregnant women has been studied on several occasions in the past: studies of the prenatal stress during the 1999 bombing in Belgrade and the armed conflict of 2011 in Libya demonstrated a significant rise in the rate of preterm deliveries, low-birth-weight infants, and cesarean sections. The authors of both studies concluded that psychosocial stress during pregnancy might cause negative pregnancy outcomes.^2^,^7^ Wainstock et al. demonstrated a higher rate of preterm deliveries among Israeli women residing close to the Gaza strip who as a consequence were constantly exposed to rocket attack during pregnancy.^4^

In July 2014 the military operation “Protective Edge” was launched by Israel in order to stop rocket attacks, which were targeting Israeli civilians.^8^ During the 51 days of the conflict (from July 7 to August 26, 2014), terrorist organizations fired thousands of rockets and mortars at Israel’s civilian population, at ranges that threatened most of the country.^9^ As detailed later, the rocket attacks were preceded by loud, sudden, and stress-inducing sirens, informing residents to seek shelter within only a few seconds before rockets hit the town. More than 4,600 sirens were sounded during 51 days of conflict, with an average of about 90 sirens per day.

The conflict led to a dramatic increase in the rate of mental health disorders, including anxiety, post-traumatic stress disorders (PTSD), depression, and other psychiatric disorders.^10^

The unfortunate situation in Israel during July–August 2014 has presented an opportunity: our aim was to study the association between exposure to stress during pregnancy and poor pregnancy outcome.

## MATERIAL AND METHODS

This was a retrospective, population-based, study. We reviewed the pregnancy outcome of all patients who during pregnancy were exposed to the stress of the military operation and delivered at the Wolfson Medical Center between July 7, 2014 and April 30, 2015—the exposed group. This medical center is in the city of Holon (in the center of Israel), and is about 70 kilometers from the Gaza strip. During the “Protective Edge” operation this region was attacked by population-targeted missiles, for the first time in its history, almost on a daily basis. During this period of time all the people in this area had to run into a shelter, in less than 30 seconds, when the alarm sounded. They were also exposed to explosive sounds and direct rockets hits. Even though most civilians were not physically hit, they suffered from constant anxiety and had abrupt stress attacks. We compared the exposed population in this area to an unexposed population: all women who delivered at the Wolfson Medical Center in the same calendar period of the year before (between July 7, 2013 and April 30, 2014); this group was defined as the unexposed group.

The medical records of all participants were retrieved from the computerized database. We reviewed maternal characteristics: age, body mass index (BMI), and parity. We checked if pregnancy was spontaneous or by assisted reproductive treatment and if it was single or multiple gestation. The following were also compared: mean gestational age (GA) at delivery and the mode of delivery; maternal chronic diseases (chronic hypertension and pre-gestational diabetes mellitus); pregnancy complications (early or late preterm delivery, defined as delivering before 34 or 37 complete weeks of gestation, respectively); gestational hypertensive disorders (which included eclampsia, preeclampsia, and pregnancy-induced hypertension); gestational diabetes mellitus; neonatal outcome and complications (birth-weight in grams, intrauterine growth restriction [IUGR], and intrauterine fetal demise [IUFD]).

Chronic hypertension and gestational hypertensive disorders were diagnosed according to the American College of Obstetrics and Gynecology recommendations.^11^ Gestational diabetes mellitus was diagnosed when a 100-g oral glucose tolerance test (OGTT) resulted in two or more pathological values, after 24 weeks of gestation. Intrauterine growth restriction was defined as birth-weight less than the 10th percentile, according to national birth-weight curves (Dolberg’s birth-weight standards).

Data were analyzed by SPSS software, version 23 (IBM, Inc.). Continuous data were compared by Student’s *t* test, and categorical data were compared by chi-square test or by Fisher’s exact test, as appropriate. Multivariate logistic regression was performed to adjust for potential confounders. A *P* value of <0.05 was considered statistically significant. The study was approved by the local Institutional Review Board.

## RESULTS

There were 4,673 women who were pregnant during the “Protective Edge” operation and delivered at the Wolfson Medical Center (exposed group), and 4,735 women who delivered in the previous year, at the same medical center (unexposed group). Compared to the unexposed group, the exposed women were slightly older (30.2±6.2 years versus 29.9±6.2 years, respectively; *P*=0.027), and had a lower rate of nulliparity (33.0% versus 36.0%, respectively; *P*=0.002). They also had higher rate of chronic hypertension (0.8% versus 0.1%, respectively; *P*<0.001), pre-gestational diabetes mellitus (1.9% versus 0.3%, respectively; *P*<0.001), and multiple gestation (4.3% versus 2.3%, respectively; *P*<0.001). The mean BMI was not significantly different between the groups. Table 1 presents maternal and pregnancy characteristics.

**Table 1:** Maternal and Pregnancy Characteristics.

	Unexposed (*n*=4735)	Exposed (*n*=4673)	*P* Value
Maternal age	29.9±6.2	30.2±6.2	0.027
BMI	23.4±4.5	23.4±4.5	0.98
Nulliparity	1703 (36.0)	1541 (33.0)	0.002
Multiple gestation	110 (2.3)	201 (4.3)	<0.001
Chronic HTN	6 (0.1)	36 (0.8)	<0.001
DM	15 (0.3)	89 (1.9)	<0.001

Data are presented as *n* (%) or mean±SD.

BMI, body mass index; DM, diabetes mellitus; HTN, hypertension.

Rates of pregnancy complications did not differ between the groups, including gestational hypertensive disorders, gestational diabetes mellitus, early or late preterm delivery, IUGR, and IUFD. Table 2 presents pregnancy complications.

**Table 2:** Pregnancy Complications.

	Unexposed (*n*=4735)	Exposed (*n*=4673)	*P* Value
GHTN	112 (2.4)	133 (2.8)	0.143
GDM	241 (5.1)	276 (5.9)	0.082
Deliveries <37 weeks	321 (6.8)	320 (6.8)	0.89
Deliveries <34 weeks	80 (1.7)	79 (1.7)	0.99
IUGR (<10%)	321 (6.94)	343 (7.5)	0.3
IUFD	15 (0.3)	21 (0.4)	0.29

Data are presented as *n* (%).

GDM, gestational diabetes mellitus; GHTN, gestational hypertensive disorders; IUFD, intrauterine fetal demise; IUGR, intrauterine growth restriction.

Regarding delivery outcome, we found a lower rate of vacuum extraction among the exposed women (4.1% versus 5%, respectively; *P*=0.02), but no differences in rates of normal deliveries or cesarean sections. There were also no differences between groups in rates of spontaneous deliveries or delivery inductions, and mean gestational age at delivery. Table 3 presents delivery outcomes.

**Table 3:** Delivery Outcomes.

	Unexposed (*n*=4735)	Exposed (*n*=4673)	*P* Value
Mean GA at delivery (weeks)	39.2±2.1	39.2±2.2	0.24
Normal vaginal delivery	3518 (74.3)	3507 (75.0)	0.4
Vacuum extraction	239 (5.0)	190 (4.1)	0.02
Cesarean section	978 (20.7)	976 (20.9)	0.78
Spontaneous onset of delivery	3404 (72.6)	3275 (70.1)	0.06
Induction of labor	739 (15.8)	786 (16.8)	0.11

Data are presented as *n* (%) or mean±SD.

GA, gestational age.

A higher percentage of male newborns was demonstrated in the exposed group (49.27% versus 45.96%, respectively; *P*=0.001). Other neonatal outcomes did not significantly differ between groups, including mean birth-weight, rate of fetal macrosomia (birth-weight of more than 4,000 g), and low birth-weight (less than 2,500 g). Table 4 presents neonatal outcomes.

**Table 4:** Neonatal Outcomes.

	Unexposed (*n*=4735)	Exposed (*n*=4673)	*P* Value
Neonatal weight, g	3222±518	3228±524	0.61
Neonatal male gender	2228 (45.96)	2402 (49.27)	0.001
Neonatal weight ≥4000 g	236 (4.86)	246 (5.04)	0.53
Neonatal weight ≤2500 g	108 (2.22)	98 (2.01)	0.54

Data are presented as *n* (%) or mean±SD.

Subgroup analyses according to the fetus’s gender, and according to gestational age (first, second, or third trimester) at the time of exposure to stress, have failed to reveal further differences between the groups in rates of pregnancy complications.

## DISCUSSION

In contrast to ours, previous studies investigating the obstetrical implications of armed conflict have found an association between mental stress during pregnancy and pregnancy complications.^2^,^4^,^7^ Bodalal et al. compared deliveries during the 2011 armed conflict in Libya to deliveries that preceded it, and found higher rates of preterm deliveries, cesarean sections, and low birth-weight (less than 2,500 g).^7^ These findings are partially contradicted by Maric et al. who studied the pregnancy outcomes among women who were exposed to the 1999 bombing in Serbia. Although that study demonstrated lower birth-weight (by 86 g) among exposed women, it also showed lower rates of cesarean sections, and no differences in rates of preterm deliveries or other pregnancy complications between exposed and unexposed groups.^2^ Wainstock et al. compared Israeli populations from two different cities (the first was subjected to constant rocket attacks, while the second was not exposed to those attacks) and found higher rates of preterm deliveries and low birth-weight (less than 2,500 g) among the exposed women.^4^ Although these studies found a correlation between low birth-weight and stress exposure, further analysis of these studies, and adjustment of their findings according to GA at birth, fails to show higher rates of IUGR among exposed women. In addition, the lack of uniformity between the study and the control groups, regarding background details and seasonality, might confound the results.^12^

Two other studies demonstrated that psychological stress during pregnancy was associated with an increased risk of stillbirth, preeclampsia, and elevated blood pressure,^13^,^14^ but neither of them had studied the effect of war-induced stress on pregnant women. Based on the HPA theory, the physiologic response to stress, which results in activation of the HPA axis, might lead to an increased blood glucose level. However, we were not able to show a higher rate of gestational diabetes among exposed women.

In our study, we compare the exposed population to women who lived in the same region and gave birth in the same calendar period of the year, and by that means we eliminated possible confounders, such as demographic details and season of the year.

The fact that we found no difference in pregnancy complication rates at all could be explained by two options: either by “too low a level of mental stress” caused by this military operation, or by the strong, rough, and durable personality of the Israeli civilians.

The literature about if and how to measure stress exposure objectively is controversial: while some studies used questionnaires or interviews in order to quantify the perceived level of stress, others defined the level of stress according to the extent of exposure, and did not evaluate the subjective stress level.^15^–^19^ Wainstock et al., who studied pregnancy complications among Israeli women who were constantly exposed to rocket attacks, found a significant correlation between the level of stress as assessed by questionnaires and the reported stress level associated with exposure to alarms.^20^ Based on this assertion, we can confidently assume that the studied population experienced different levels of stress.

This study has several strengths: it is one of the largest studies ever conducted about the association between prenatal mental stress and pregnancy complications among a population subjected to military attacks. In addition, despite the complexity in conducting this kind of study, the careful selection of the control group enabled us to isolate the studied parameters and to consider the effect of possible confounders as negligible. There are two limitations to this study: first, its retrospective design; second, the lack of a second control—i.e. women who conceived after the military operation and delivered during the following year.

In conclusion, exposure to military-induced mental stress during pregnancy, for a period of almost two months, has not been found to be associated with increased risk for pregnancy complications, regardless of the extent of stress or the gestational age at the time of exposure.

## Abbreviations

BMI: body mass index
GA: gestational age
HPA: hypothalamic–pituitary–adrenal
IUFD: intrauterine fetal demise
IUGR: intrauterine growth restriction
